# The seroprevalence of HIV in patients undergoing lower limb Total Joint Arthroplasty (TJA) in South Africa

**DOI:** 10.1051/sicotj/2019042

**Published:** 2020-01-22

**Authors:** Zia Maharaj, Jurek Rafal Tomasz Pietrzak, Nkhodiseni Sikhauli, Dick van de Jagt, Lipalo Mokete

**Affiliations:** 1 Charlotte Maxeke Johannesburg Academic Hospital Jubilee Road, Parktown Johannesburg Gauteng 2196 South Africa

**Keywords:** HIV/AIDS, Total Joint Arthroplasty, Total Hip Arthroplasty, Total Knee Arthroplasty

## Abstract

*Aim*: The aim was to assess the seroprevalence of Human Immunodeficiency Virus (HIV) in non-haemophilic patients undergoing primary Total Joint Arthroplasty (TJA) at an academic hospital in South Africa.

*Methods*: A retrospective review of all Total Hip Arthroplasty (THA) and Total Knee Arthroplasty (TKA) patients from January 2017 to December 2018 was conducted. All patients awaiting TJA were offered HIV screening and their demographic data were recorded. Consenting patients were tested or the refusal of testing was documented. The CD4+ T-cell count (CD4+) and viral load (VL) was measured for all HIV-positive patients and newly diagnosed patients were initiated on Highly Active Antiretroviral Treatment (HAART).

*Results*: We included 1007 patients in the study. The TJA population HIV seroprevalence was 10.7% (*n* = 108). The seroprevalence for THA was 14.9% (*n* = 78) and that for TKA was 6.2% (*n* = 30). There were 93 patients (9.2%) who refused screening. There were 12 (15.4%) and 3 patients (10%) that were newly diagnosed in the THA and TKA seropositive populations, respectively. The average CD4+ for THA and TKA was 569 cells/mm^3^ (105–1320) and 691 cells/mm^3^ (98–1406), respectively. The VL was undetectable in 75.9% (*n* = 82) of HIV-positive patients. Overall 12 HIV-positive patients (11.12%) had CD4+ <200 cells/mm^3^, 8 of these patients (66%) were newly diagnosed. The average age of the seropositive population was 58 ± 6.5 years and 66 ± 8.5 years for THA and TKA, respectively (*p* = 0.03). Femoral head osteonecrosis was the underlying pathology for 65.38% (*n* = 51) of seropositive patients for THA.

*Conclusion*: The seroprevalence of HIV in patients undergoing THA in our South African institution is greater than the seroprevalence in the general population. The seroprevalence of HIV in THA is significantly greater than that in TKA. This may reflect the association between HIV, HAART and hip joint degeneration. Our findings draw attention to the significant burden HIV has on TJA.

## Introduction

Total Joint Arthroplasty (TJA) is a commonly performed orthopaedic procedure with advances in implants and surgical techniques producing excellent outcomes in the majority of patients [1]. The demand for TJA is high with over 1.4 million and 131 005 operations performed annually in the United States of America (USA) [2] and Canada [3], respectively [4]. There were approximately 91 698 Total Hip Arthroplasty (THA) and 102 177 Total Knee Arthroplasty (TKA) procedures performed in England and Wales in 2017 [5]. The projected increase in demand is 146% for THA and 768% for TKA, respectively, between 2010 and 2030 [4]. With cost per single procedure ranging between US$ 16 000 and US$ 60 000 [6], the increase in popularity of TJA places significant economic implications on the healthcare system.

Human Immunodeficiency Virus (HIV) is a pandemic affecting more than 36.9 million people worldwide [7]. South Africa accounts for the most people living with HIV (7.2 million) worldwide, and the highest cost burden of US$ 2 073 272 539 for patient management [7]. This is further compounded by the highest rate of new infections with 270 000 new diagnoses reported in 2017 alone [7]. However, HIV remains of global public health concern with over 2 million people living with HIV in the European Region and 159 420 new diagnoses made in 2017 [8]. Despite the significantly lower HIV prevalence rates, there are an estimated 1.1 million people and 120 000 people living with HIV in the USA [9] and United Kingdom [10] respectively. There is still no known cure for HIV and patient management is centered around disease control with Highly Active Anti-retroviral Treatment (HAART) [11].

The prevalence of HIV-infected patients undergoing TJA is increasing worldwide [6]. Remarkably, this trend has even been observed in regions with the lowest incidence of HIV [12]. Analysis of the USA Nationwide Inpatient Sample for the years 2000 through 2008 noted a significant increase in HIV-infected patients presenting for primary TJA (*p* < 0.05) [12]. There are multiple factors that contribute to the role that HIV-infection may have on patients for TJA. Firstly, widespread improvements in access to HAART have resulted in HIV-positive patients being able to expect normal life-spans [7] and subsequently develop chronic diseases including degenerative joint conditions. Additionally, HIV-infected patients may be at a greater risk for osteodegenerative pathology, as both HIV and HAART are associated with an increased incidence of osteonecrosis and osteoporosis [13, 14]. HIV-positive patients have a 45-to-100-fold increased risk of developing avascular necrosis (AVN) of the femoral head compared to the general population [15]. Furthermore, the incidence of AVN has increased since the advent of HAART [13]. Several of the HAART pharmaceutical agents have been reported to independently decrease Bone Mineral Density (BMD) [14]. The resultant osteoporosis predisposes HIV-positive patients to Neck of Femur (NOF) Fractures [13]. HIV-positive patients are both 3.7 times more likely to be osteoporotic [14] and sustain more low-trauma fractures [13] when compared to HIV-negative controls.

Recent literature reports equivocal functional outcomes after TJA in HIV-positive patients controlled on HAART when compared to HIV-negative controls [15, 16]. However, several studies indicate that immunocompromised patients may be predisposed to post-operative complications [15]. A systematic review of TJA outcomes for over 6.5 million procedures by Dimitriou et al. [15] indicates that HIV-positive patients have a significantly increased risk of peri-Prosthetic Joint Infections (PJI), at 7.6% compared to HIV-negative controls, at 3.3%. Similarly, a retrospective review of approximately 28 000 patients aimed to evaluate pre-operative risks for PJI after THA concluded that HIV-infection was amongst the most influential risk factors and should be considered when counselling patients for surgery [17]. The 2018 International Consensus on Orthopaedic Infections determined that although HIV is an independent risk for PJI, this significance is negated once HAART is initiated and viral suppression is achieved [18].

Several studies have suggested that HIV screening before elective orthopaedic surgery, especially for patients with risk factors, may be beneficial to surgeons [19, 20]. Amongst healthcare workers, surgeons have the highest incidence of exposure reported as approximately 12 percutaneous blood exposures per person per year [21]. Transmission risk is increased exponentially with increased VLs which is found in patients who remain undiagnosed and not yet initiated on HAART [11]. Many studies have described the outcomes of TJA in HIV-positive patients [15, 16]; however, none have quantified the seroprevalence of HIV in patients presenting for TJA in South Africa.

The aim of this study was to determine the seroprevalence of HIV in non-haemophilic patients undergoing TJA, at a single sub-saharan urban academic institution between January 2017 and December 2018. Secondarily, we sought to investigate the differences in the seroprevalence of THA compared to TKA. Lastly, we assessed the status of disease control and primary joint pathology of seropositive patients.

## Patients and methods

This study was an observational retrospective review of all patients presenting for TJA to the Orthopaedic Arthroplasty Unit at Charlotte Maxeke Johannesburg Academic Hospital (CMJAH) in Gauteng, South Africa, between January 2017 and December 2018. This is a quaternary referral centre serving patients from surrounding provinces and neighbouring sub-Saharan countries. Demographic data of patients were recorded including age, Body Mass Index (BMI), gender, joint side affected, medical co-morbidities and American Society of Anesthesiologists Classification (ASA Class). Patients eligible for TJA were offered Voluntary Counselling and Testing (VCT) to screen for HIV, as per national ethical protocol [11]. Consenting patients were tested for HIV whilst patient refusal to test despite adequate counselling was recorded. Subjects were included in the study if they were 18 years of age or older, underwent TJA during this 24-month time frame and received VCT. Subjects were excluded if the HIV status was unknown with no documented evidence of refusal to screen, which may indicate that VCT was not offered. Serum markers of disease status, namely CD4+ and VL, were measured for all HIV-positive patients. Newly diagnosed seropositive patients were initiated on HAART regardless of CD4+ in line with the World Health Organization (WHO) 2015 recommendations [11].

The National Health Laboratory Services of South Africa performed the blood tests measured in the study. For patients who consented to HIV screening a standard HIV fourth generation rapid strip antigen test was initially performed. A positive or indeterminate result was confirmed with an HIV fourth generation Enzyme-Linked Immunosorbent Assay (ELISA) [22]. The HIV-1 RNA Polymerase Chain Reaction (PCR) VL ultra-sensitive assay reports an undetectable result as lower than the detectable limit (LDL) of 50 copies/mL [22]. All blood tests are FDA-approved in the USA and have adequate sensitivity as per current global methods available [22].

The most recent pre-operative radiographs of seropositive patients for TJA were evaluated to determine joint pathology. Senior authors, JRTP and LM, reviewed the radiographs and diagnosis was made on gross radiological appearance. The two authors evaluated the pelvis anteroposterior (AP) and lateral hip radiographs for patients undergoing THA and AP, lateral and skyline views of both knees in those presenting for TKA.

All continuous variables were analysed using *t*-tests. Subgroup analysis was performed for statistical analysis, the study population was divided into groups for THA and TKA. Standard descriptive statistics including chi-squared tests and unadjusted logistic regression were used to compare seroprevalence between THA and TKA groups. Risk adjusted differences were compared using multivariable logical regression adjusting for underlying differences in age, gender, BMI and ASA class. All analyses of data were performed using Stata Statistical Software: Release 13.0 (StataCorp LP, College Station, TX). Statistically significant two-sided *p* values were defined as *p* < 0.05.

## Results

**Figure 1:**
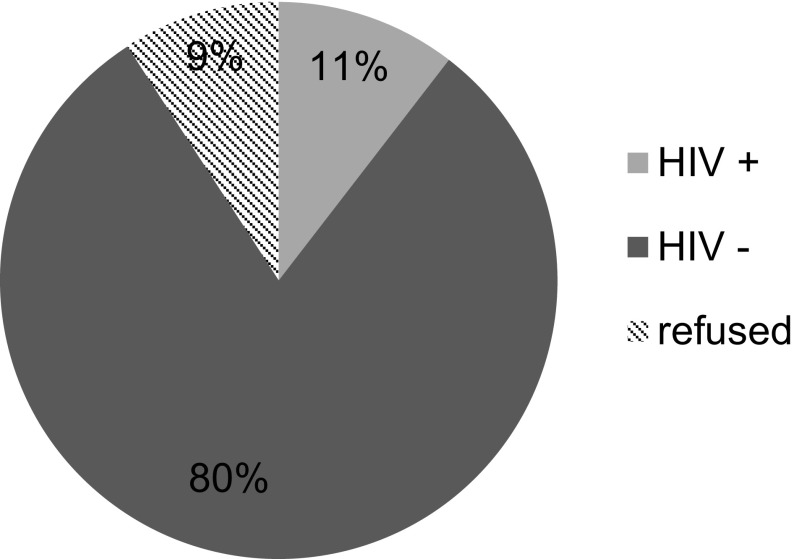
Total joint arthroplasty population voluntary counselling and testing results.

**Figure 2:**
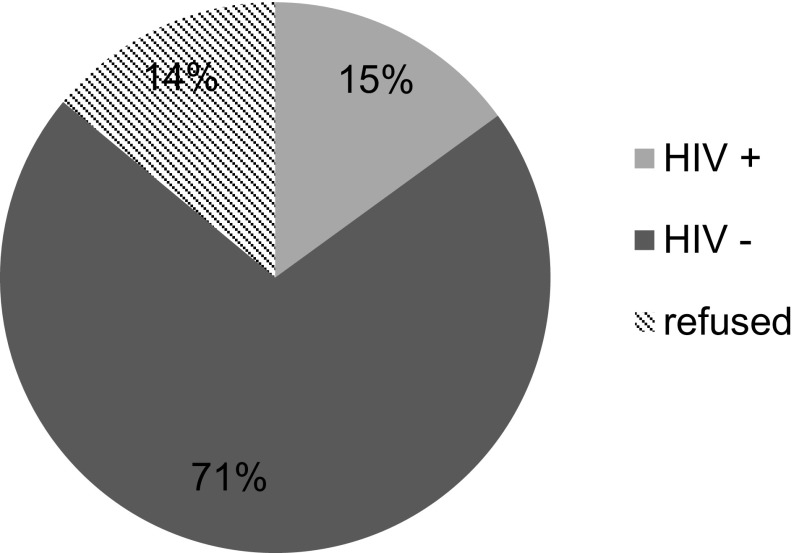
Total hip arthroplasty population voluntary counselling and testing results.

**Figure 3:**
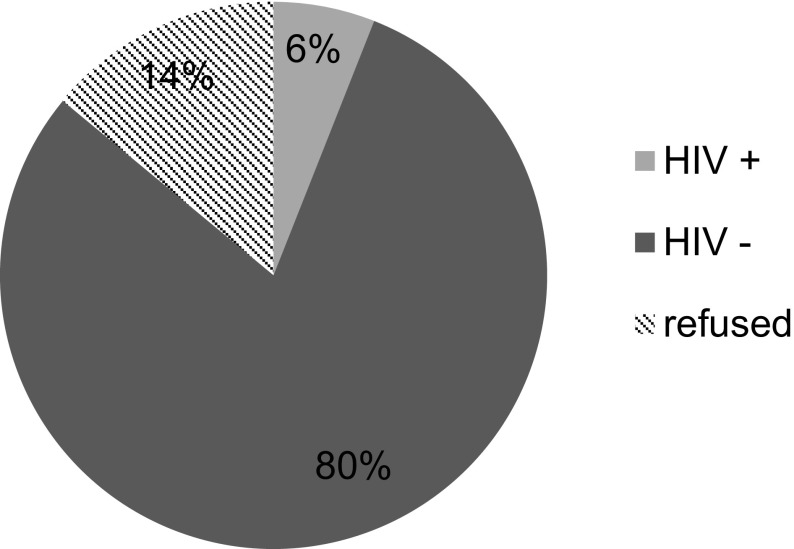
Total knee arthroplasty population voluntary counselling and testing results.

There were 1052 patients who presented for TJA during the study time frame. Neither blood test results of HIV status nor evidence of refusal to screen could be traced in 45 patients (4.28%) and these patients were excluded from the study. Subsequently the results of 1007 patients were included in this study. There were 524 (52%) patients 483 (48%) patients who underwent THA and TKA, respectively. Overall, 914 patients (90.8%) consented to HIV screening. There were 108 HIV-positive patients (10.7%) in the total TJA population (Figure 1). This was subdivided into 14.9% (*n* = 78) for THA and 6.2% (*n* = 30) for TKA as shown in Figures 2 and 3, respectively. There were 93 patients (9.2%) who refused voluntary screening despite adequate counselling. There were 12 patients (15.4%) and 3 patients (10%) newly diagnosed in the THA and TKA seropositive populations respectively and initiated on HAART. The 93 HIV-positive patients with known status (86.1%) were on pre-existing HAART.

The average age of the HIV-positive and HIV-negative patients for THA was 58 ± 6.5 years and 64 ± 7.3 years, respectively (*p* = 0.04) (Table 1). The BMI of seropositive and seronegative patients for THA was 33.43 kg/m^2^ and 27.65 kg/m^2^, respectively (*p* = 0.045). There were no statistically significant findings in the TKA population. The average age of the seropositive population was 58 ± 6.5 years and 66 ± 8.5 years for THA and TKA, respectively (*p* = 0.03). The BMI was 27.65 kg/m^2^ and 30.76 kg/m^2^ in seropositive patients for THA and TKA, respectively (*p* = 0.021) (Table 2).

**Table 1:** Demographic results of total joint arthroplasty population,

	Total hip arthroplasty				Total knee arthroplasty			
	HIV −ve	HIV +ve	N/A	*p*-value	HIV −ve	HIV +ve	N/A	*p*-value
Number of patients	395	78	51		411	30	42	
Age	64 ± 7.3	58 ± 6.5	62 ± 7.4	0.04	67 ± 9.1	66 ± 8.5	64 ± 8.47	0.9
Male/Female	97/298	51/27	6/45	0.84	40/371	16/14	9/33	0.9
Joint side (Left/Right)	186/209	42/36	22/29	1.00	226/185	12/18	11/31	0.086
ASA Class	2 (1–3)	2 (1–3)	2 (1–3)	1.00	2 (1–3)	2 (1–3)	2 (1–3)	1.00
BMI (kg/m^2^)	33.43 ± 8.6	27.65 ± 7.3	30.23 ± 7.54	0.045	34.82 ± 6.8	30.76 ± 7.9	31.67 ± 6.2	0.07

HIV –ve, HIV negative; HIV +ve, HIV positive; N/A, Not Available (unknown HIV status); ASA Class, American Society of Anesthesiologists Classification; BMI, Body Mass Index.

**Table 2:** Demographic results of HIV-positive total joint arthroplasty population.

	Total hip arthroplasty	Total knee arthroplasty	*p*-value
Number of patients	78	30	
Age	58 ± 6.5	66 ± 8.5	0.03
Male/Female	51/27	16/14	0.076
Joint Side (Left/Right)	42/36	12/18	1.00
ASA Class	2 (1–3)	2 (1–3)	1.00
BMI (kg/m^2^)	27.65 ± 7.3	30.76 ± 7.9	0.021

ASA Class, American Society of Anesthesiologists Classification; BMI, Body Mass Index.

**Figure 4:**
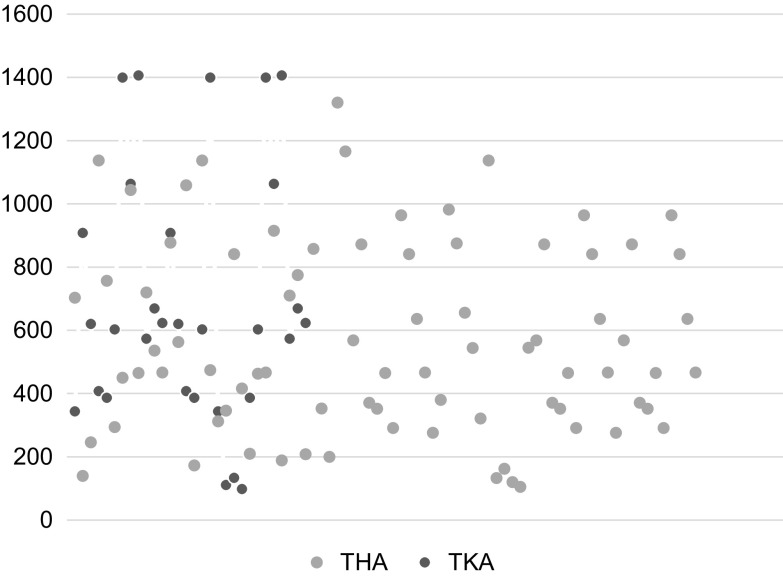
Scatter plot of CD4+ T-cell count in HIV-positive total joint arthroplasty population.

The average CD4+ for THA and TKA was 569 cells/mm^3^ (105–1320) and 691 cells/mm^3^ (98–1406), respectively (Figure 4). Overall, 12 HIV-positive patients (11.12%) had CD4+ <200 cells/mm^3^, 8 of these patients (66%) were newly diagnosed. The VL was undetectable in 75.9% (*n* = 82) of HIV-positive patients. Femoral head AVN and NOF was the underlying pathology in 65.38% (*n* = 51) and 7.7% (*n* = 6) of seropositive patients for THA, respectively (Table 3). Primary osteoarthritis was the pathology in 60% (*n* = 18) of seropositive patients for TKA and there were no cases that presented with a diagnosis of AVN of the knee.

**Table 3:** Pathology of seropositive patients for total joint arthroplasty.

Joint affected	Hip		Knee	
Pathology	Number of patients	Percentage of total (%)	Number of patients	Percentage of total (%)
Avascular necrosis	51	65.38	0	0
Primary osteoarthritis	14	17.95	18	60
Secondary Osteoarthritis	7	8.97	12	40
Inflammatory	7	8.97	7	23.33
Post-traumatic	0	0	3	10
Tuberculosis	0	0	2	6.67
Neck of Femur fracture	6	7.7	N/A	N/A

## Discussion

The HIV seroprevalence of patients for primary TJA at our institution was 10.8%. Several international studies have reported the seroprevalence of HIV in patients for orthopaedic surgery (Table 4). Only three other studies have screened for HIV in patients for TJA between 1993 and 2019 [12, 19, 23]. There have been no other studies conducted in Africa designed to screen for HIV in a TJA population for comparison; however, there have been two reports incidentally indicating HIV prevalence [24, 25]. A study conducted in Malawi by Lubega et al. (2009) [24] found a HIV seroprevalence of 33% in 42 patients screened (out of 58 total patients) who underwent THA in Malawi. Mulla et al. (2010) [25] reported similar seroprevalence findings of one-third in their retrospective review conducted in Zambia; however, only 12% of patients for TJA were screened for HIV. To our knowledge, this is the first study conducted in sub-Saharan Africa to evaluate the seroprevalence of patients awaiting TJA.

**Table 4:** Seroprevalence of HIV in patients for orthopaedic surgery.

Study	Country	Number of patients	Population*	Seroprevalence
Miniero et al. (1997) [20]	Portugal	288	General	1.7
Sanchez et al. (1998) [30]	Puerto Rico	100	Trauma	7
Sefeane et al. (2011) [26]	South Africa	261	Trauma	23
Yeganeh et al. (2015) [31]	Iran	320	Trauma	0.6
Lin et al. (2013) [12]	United States of America	5 681 024	TJA	0.14
Winkelmann et al. (2016) [19]	Germany	1534	TJA	0.06
Cheng et al. (2017) [23]	China	11 609	TJA	0.08
Our study	South Africa	1007	TJA	10.8

* Refers to specific patient population included in study. General, entire orthopaedic department; Trauma, orthopaedic trauma; TJA, Total Joint Arthroplasty.

A previous study of patients undergoing orthopaedic trauma surgery in the same South African institution by Sefeane et al. (2011), reported an HIV seroprevalence of 23% [26]. The higher seroprevalence in trauma surgery patients when compared to those for TJA may in part be as a consequence of the age group most commonly affected. The highest risk population for orthopaedic trauma is reported to be 20–49 years [26], whilst concurrently the highest seroprevalence for adults in South Africa affects those aged 15–49 years [7]. In contrast a much older demographic presents for TJA, with the mean age of patients for THA and TKA stated as 68 years and 69 years, respectively [5].

The seroprevalence of HIV in the patients for THA (14.9%) was significantly higher than those for TKA (6.2%). There were 108 seropositive patients for TJA in our study, of which 72% (*n* = 70) were undergoing THA. The predominant indication in the seropositive patients for THA was femoral head AVN (65.4%). The average age of seropositive patients for THA was lower than those for TKA, 58 years compared with 66 years, respectively (*p* = 0.03). The average BMI of patients for THA (27.65 kg/m^2^) was lower than those for TKA (30.76 kg/m^2^), (*p* = 0.021). Remarkably, some similar trends were noted for the Nationwide Inpatient Sample in the USA including approximately 5.7 million patients undergoing TJA with an HIV seroprevalence of 0.14% [12]. Lin et al. (2013) reported that of all HIV-positive patients for TJA, 79% (*n* = 6499) were undergoing THA [12]. Furthermore, after analysis comparing patient characteristics with HIV-negative controls, Lin et al. (2013) concluded that HIV-infected patients were more likely to be male, younger, and have a history of femoral head AVN [12]. These findings are in keeping with current evidence describing the high risk of osteodegeneration associated with HIV and HAART [6, 13]. Additionally, literature has indicated that HIV-positive patients suffer osteonecrosis of the hip at a younger age than HIV-negative controls and present for THA after a mean of 25 months post symptom onset as compared with 4 years, respectively [27].

The serum markers of disease status, namely CD4+ and VL, indicated that most seropositive patients were controlled on HAART. VL is a reliable marker of treatment efficacy and patients adherent to HAART should have an undetectable VL [11]. The VL was undetectable in 75.9% (*n* = 82) of HIV-positive patients which is likely due to the fact that 86.1% of patients were on pre-existing HAART. The CD4+ is a surrogate marker of immune status and patients with CD4+ <200 cells/mm^3^ have an increased morbidity and mortality due to opportunistic infections [28]. There were 12 patients (15.4%) newly diagnosed in the THA population with the implementation of routine screening. Approximately two-thirds (66%) of those newly diagnosed were immunocompromised which may contribute to an increased risk of PJI after TJA [15]. This highlights the possibility of an increased demand in undiagnosed HIV-positive patients for THA, not only in the high risk South African population but worldwide.

This study had several limitations. Firstly, the ethical protocol of VCT in South Africa must be addressed. It is mandatory that patients are counselled, and provide consent prior to HIV screening due to the lifelong implications of a positive-diagnosis. Despite the implementation of access to free VCT and HAART, a negative stigma amongst South Africans observed by patients refusing to be screened may exist. Despite adequate counselling, we failed to get consent for HIV testing in 93 patients (9.2%). This may be as a consequence of being poorly educated on the improved outcomes for patients adherent to HAART and risks of late diagnosis and delayed initiation of treatment. However, the VCT protocol is specific to South Africa and does not necessarily apply in other countries. An additional limitation of the study was due to clerical error whereby 45 patients (4.28%) missed the screening opportunity. We attribute this error to the academic institution setting whereby there are frequent rotations of junior staff within the department. There was no formal handout prescribed to new staff members upon induction into the unit during the time of study conception and it became apparent that they were not being adequately informed of the mandatory offer of VCT for our TJA patients. We subsequently compiled a written protocol and the number of missed opportunities decreased as a result.

The seroprevalence of HIV in the THA population (14.9%) was significantly higher than the TKA population (6.2%) and the reported average in the general population of South Africa (12.6%) [29]. The demand for TJA in HIV-positive patients is increasing worldwide and the association between THA and HIV is undeniable. HIV should be excluded in young patients presenting for THA with unexplained femoral head AVN and low energy NOF fractures. We recommend routine HIV screening as protocol in any setting for all patients presenting for THA, particularly in population groups with high HIV infection rates.

## Declarations

### Plagiarism

- The authors confirm that the work submitted is original and does not transgress the plagiarism policy of the journal.
- No data, text or theories by others are presented as if they were the author’s own.
- Proper acknowledgements of other’s work has been given, quotation marks are used for verbatim copying of material.
- Permissions have been secured for material that is copyrighted.
